# *Prasiolopsis wulf-kochii* (Prasiolales, Trebouxiophyceae), a New Species Occurring in Hairs of the Sloth *Bradypus tridactylus*

**DOI:** 10.3390/plants13172405

**Published:** 2024-08-28

**Authors:** Tatyana Darienko, Thomas Pröschold

**Affiliations:** 1 Research Department for Limnology, Leopold-Franzens-University of Innsbruck, A-5310 Mondsee, Austria; tdarien@gwdg.de; 2 Institute for Microbiology and Genetics, Department of Applied Bioinformatics, Georg-August-University of Göttingen, D-37077 Göttingen, Germany

**Keywords:** ITS-2/CBC approach, ITS secondary structures, morphology, phylogeny, *Prasiolopsis ramosa*, SSU, *Trichophilus welckeri*

## Abstract

The monotypic genus *Prasiolopsis* has been known for a long time, but is often overlooked because of difficulties in identification and the morphological variability between uniseriate filaments and cell packages forming pseudoparenchymatic thalli depending on age. We investigated a strain (SAG 84.81) originally denoted as *Trichophilus welckeri*, which was isolated from the hairs of the sloth *Bradypus tridactylus,* and compared it with other available strains of *Prasiolopsis* and of the sister genus *Pseudomarvania*. Our investigations clearly showed that this strain differed in morphology, especially of the chloroplast, from those originally described for *Trichophilus*. Phylogenetic analyses of the SSU and ITS rDNA sequences revealed that the strain SAG 84.81 is sister to several strains of *P. ramosa* within the *Prasiola* clade (Trebouxiophyceae). Using the ITS-2/CBC approach, we clearly demonstrated that this strain represented a new species of *Prasiolopsis*, which we proposed here as *P. wulf-kochii*. In addition, we evaluated the ITS-2/CBC approach by comparing it with the two species of *Pseudomarvania*. All investigated strains showed CBCs and HCBCs, which support their species delimitation. The sequencing data of *Trichophilus welckeri* available in GenBank were phylogenetically re-evaluated by including all representatives of the Ulotrichales (Ulvophyceae). Our analyses showed that these sequences formed their own lineage within this order.

## 1. Introduction

The genus *Prasiolopsis* was described by Vischer [1] as an alga, which was known for a long time as *Pleurococcus vulgaris* [2,3]. It is characterized by uniseriate filaments at young stages, and cell packages forming pseudoparenchymatic thalli when they become older. The presence of an asteroid chloroplast with a central pyrenoid demonstrated that this genus belongs to the order Prasiolales. Originally, this order was established by Schaffner [4], containing only the genus *Prasiola* (C. Agardh) Meneghini. Knebel [5] provided the first monograph on the Prasiolales and added the genus *Gayella* Rosenvinge, which was later renamed by Silva [6] to *Rosenvingiella*, in this order. In addition to *Prasiolopsis,* Vischer [1] described the genus *Prasiococcus*, which was found on calcified stones. The species of *Prasiola* and *Rosenvingiella* are morphologically characterized by the formation of blade-like pseudoparenchymatic thalli, which are derived from cell packages and sexual reproduction by oogamy [7,8,9,10]. They occur in marine and freshwater habitats. The monotypic genera *Prasiococcus* and *Prasiolopsis* were found in terrestrial habitats and had a sarcinoid organization. The latter genus formed irregularly branched filaments with age [1,10,11].

Ultrastructural studies have revealed that the Prasiolales have a counterclockwise orientation in the flagellar basal bodies of motile cells [12], which are typical for Trebouxiophyceae. Phylogenetic studies confirmed that all these genera belong to the so-called *Prasiola* clade of the Trebouxiophyceae [13,14,15]. Heesch et al. [16] established three new genera among the Prasiolales: *Prasionema* Heesch, Pazoutova & Rindi, *Prasionella* Heesch, Pazoutova & Rindi and *Rosenvingiellopsis* Heesch, Pazoutova & Rindi. All these representatives belong to the family Prasiolaceae Blackman & Tansley and formed a monophyletic lineage (*Prasiola* clade) within the Trebouxiophyceae, with the surprise that other genera with different morphologies (coccoid or rod-like short filaments or sarcinoid) also belonged to this group [11,17,18]. The following genera are members of the *Prasiola* clade (= Prasiolales emend.) besides the taxa already mentioned above: *Raphidonema* Lagerheim, *Pseudochlorella* J.W.G.Lund, *Edaphochlorella* Darienko & Pröschold, *Pseudomarvania* Elias & Neustupa, *Stichococcus* Nägeli, *Desmococcus* F.Brand, *Diplosphaera* Bialosuknia, *Pseudostichococcus* L.Moewus, *Protostichococcus* Pröschold & Darienko, *Deuterostichococcus* Pröschold & Darienko, *Tritostichococcus* Pröschold & Darienko and *Tetratostichococcus* Pröschold & Darienko.

Interestingly, Karsten et al. [14] sequenced the SSU rDNA of a strain (SAG 84.81) originally assigned as *Trichophilus welckeri*, which was closely related to the authentic strain of *Prasiolopsis ramosa* Vischer (SAG 26.83). The phylogenetic position of SAG 84.81 was later confirmed by plastome data [19]. *Trichophilus welckeri* was originally described by Weber-van-Bosse [20] as an alga that grew on and in the hairs of the sloth *Bradypus tridactylus*. This alga formed prostrate, irregularly branched, uniseriate to partly multiseriate filaments with uninucleate cells, each with a parietal chloroplast without a pyrenoid. *Trichophilus* reproduces using two types of motile cells, large quadri- and small biflagellated zoospores. Suutari et al. [21] investigated 71 hair samples of six sloth species (*Bradypus variegatus*, *B. tridactylus*, *B. torquatus*, *B. pygmaeus*, *Choloepus hoffmanni* and *C. didactylus*) collected in French Guiana, Panama, Costa Rica and Brazil. The sequencing results of these samples revealed that the different sloth species hosted a number of green algae as well as other pro- and eukaryotic microorganisms. However, one alga was frequently found and often abundant on sloth fur, which they concluded to be *Trichophilus welckeri* based on a morphological comparison with the original description. In contrast to the phylogenetic position of SAG 84.81, this alga did not belong to the Trebouxiophyceae; it represented a lineage within the Ulvophyceae.

The aim of this study is to compare the morphology and SSU/ITS rDNA sequences of SAG 84.81 with other strains of *Prasiolopsis ramosa* using the integrative approach used for *Stichococcus* and its relatives by Pröschold and Darienko [11]. In addition, we re-analyzed the SSU data designated as *Trichophilus welckeri* by Suutari et al. [21] to find out the correct phylogenetic position among the Ulvophyceae.

## 2. Results

### 2.1. Molecular Phylogeny of Prasiolopsis and Its Relatives

**Figure 1:**
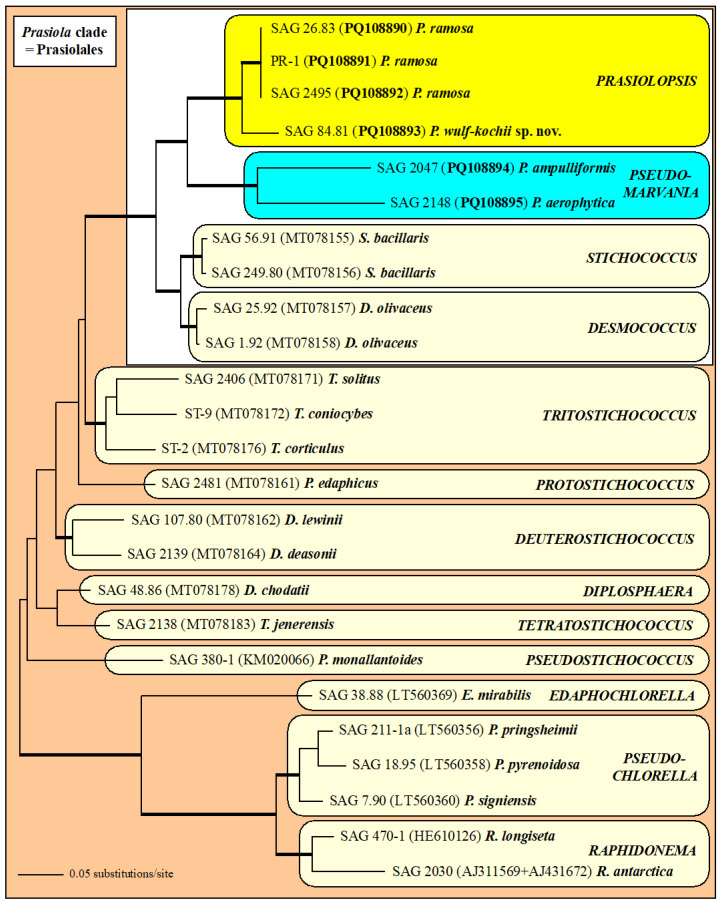
Molecular phylogeny of the *Prasiola* clade (Trebouxiophyceae) based on SSU and ITS rDNA sequence comparisons. The phylogenetic tree shown was created using the maximum likelihood method based on a data set of 2671 aligned positions of 25 taxa using PAUP 4.0a build169. For the analysis, the GTR+I+G (base frequencies: A 0.22644, C 0.29080, G 0.27799, U 0.20477; rate matrix A-C 1.7066, A-G 1.7909, A-U 1.1175, C-G 1.1230, C-U 4.3140, G-U 1.0000) with the proportion of invariable sites (I = 0.6004) and gamma-shaped parameters (G = 0.4542) was chosen, which was calculated as the best model by an automated model selection tool implemented in PAUP. The branches in bold are highly supported in all analyses (Bayesian values >0.95 calculated with PHASE and MrBayes; bootstrap values >70% calculated with PAUP using maximum likelihood, neighbor-joining, maximum parsimony and RAxML using maximum likelihood). The sister genera *Raphidonema*, *Pseudochlorella* and *Edaphochlorella* were chosen as an outgroup. The newly sequenced strains are highlighted in bold.

The phylogenetic analyses of the concatenated data set of SSU and ITS rDNA sequences revealed that the investigated strains formed two lineages within the *Prasiola* clade of the Trebouxiophyceae (Figure 1). Both the genera *Prasiolopsis* and *Pseudomarvania* (highlighted in yellow and blue in the Figure 1, respectively) are sisters to a clade consisting of the genera *Stichococcus* and *Desmococcus*. All of the genera were highly supported in all of the Bayesian and bootstrap analyses. The four strains of *Prasiolopsis* differed in 99 bases in the SSU/ITS sequences in total. The strains SAG 26.83 and SAG 2495 were completely identical and strain PR-1 only differed by one base from the other two of *Prasiolopsis ramosa*. In contrast, the strain SAG 84.81, which was originally assigned as *Trichophilus welckeri,* showed 98 base differences. 229 base differences could be observed between the two strains of *Pseudomarvania* representing two species (*P. ampulliformis* and *P. aerophytica*). All of the newly investigated strains had a group I intron at position 1506 with length variations of 447 bases in SAG 2047 up to 508 bases in SAG 84.81. Interestingly, the strain SAG 2148 had an additional intron at position 943 with an exceptional length of 1762 bases. The BLASTn search [22] for this intron did not find any similarity in GenBank.

**Figure 2:**
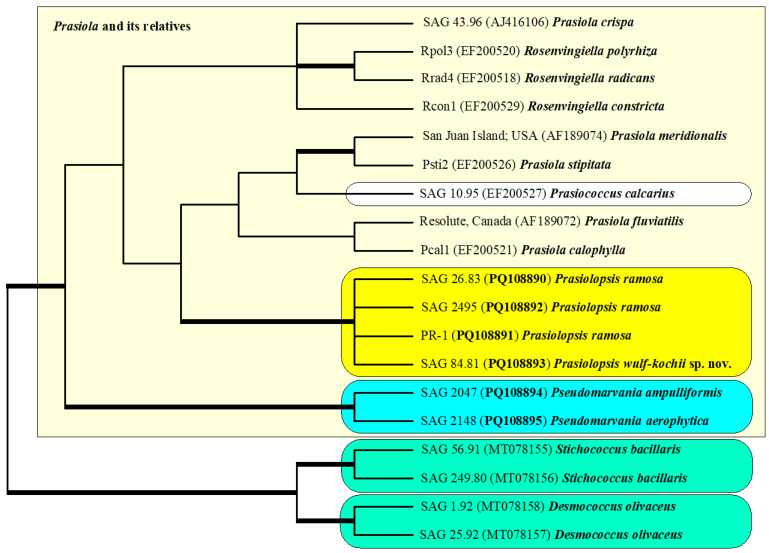
Molecular phylogeny of the *Prasiolaceae* within the *Prasiola* clade (Trebouxiophyceae) based on SSU rDNA sequence comparisons. The phylogenetic tree shown was created using the maximum likelihood method based on a data set of 1781 aligned positions of 19 taxa using PAUP 4.0a build169. For the analysis, the TIM+I+G (base frequencies: A 0.25323, C 0.21503, G 0.27496, U 0.25678; rate matrix A-C 1.0000, A-G 1.6233, A-U 0.5636, C-G 0.5636, C-U 6.2596, G-U 1.0000) with the highest proportion of invariable sites (I = 0.8541) and gamma-shaped parameters (G = 0.9731) was chosen, which was calculated as the best model by automated model selection tool implemented in PAUP. The branches in bold are highly supported in all analyses (Bayesian values >0.95 calculated with PHASE and MrBayes; bootstrap values >70% calculated with PAUP using maximum likelihood, neighbor-joining, maximum parsimony and RAxML using maximum likelihood). The sister genera *Stichococcus* and *Desmococcus* were chosen as an outgroup (marked in green). The newly sequenced strains are highlighted in bold.

The phylogenetic tree presented in Figure 1 did not contain any sequences of *Prasiola* and its related taxa because no SSU and ITS rDNA sequences are available. To find out the phylogenetic relationship between these taxa and our investigated strains (marked in a white box in Figure 1), we analyzed them with SSU rDNA sequences of representative taxa belonging to the Prasiolaceae. The phylogenetic analyses revealed that the genera *Prasiola*, *Rosenvingiella*, *Prasinococcus* and *Prasiolopsis* were sisters to the genus *Pseudomarvania* (Figure 2). Interestingly, *Prasiola* and *Rosenvingiella* seemed to be polyphyletic. However, the SSU sequences among both genera showed a low variability (~2%). The sarcinoid *Prasinococcus* is phylogenetically similar among different blade-forming *Prasiola* and *Rosenvingiella*.

The genetic differences within the strains belonging to *Prasiolopsis* and *Pseudomarvania* varied as mentioned above (99 versus 229) among the SSU and ITS rDNA sequences. Both *Pseudomarvania* strains represented species, which raised the question of whether the differences among the *Prasiolopsis* strains were also differences between species. To figure out if that is the case, we compared the secondary structures of ITS-1 and ITS-2 of both genera.

**Figure 3:**
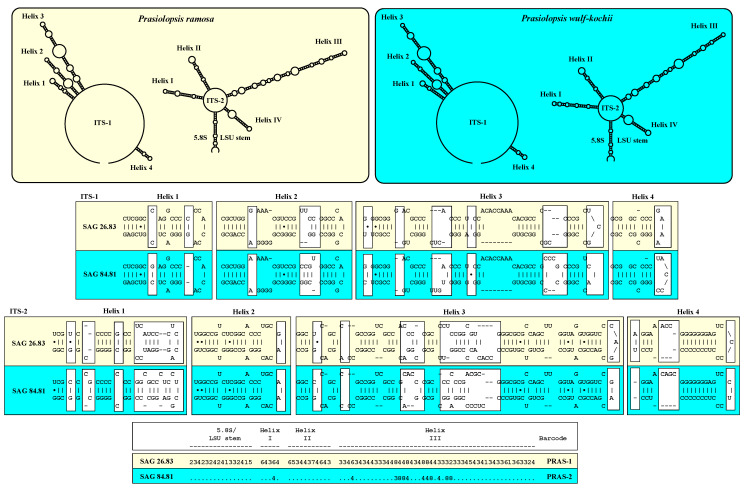
ITS-1 and ITS-2 secondary structures of the *Prasiolopsis* strains investigated in this study. The line structures have been drawn with PseudoViewer (http://pseudoviewer.inha.ac.kr). The helices were folded with mfold (http://www.unafold.org/RNA_form.php). The white boxes indicate the differences between both species. The conserved region of ITS-2 was used as barcode using the ITS-2/CBC approach as demonstrated in Pröschold and Darienko [11]. The ITS-2 Barcode is given as a number code (1 = A–U; 2 = U–A; 3 = G-C; 4 = C–G; 5 = G•U; 6 = U•G; 7 = mismatch; 8 = deletion, single or unpaired bases). The species are highlighted in the different colors.

The strains of *Prasiolopsis ramosa* (SAG 26.83, SAG 2495 and PR-1) had identical ITS-1 and ITS-2 secondary structures, but the strain SAG 84.81 differed in both ITS-1 and ITS-2, which indicated that this strain represents another species (Figure 3). The ITS-1 secondary structures of both species showed differences, especially at the end of each of the four helices. These four helix structures are typical for green algae, as presented (see Coleman and Mai [23]). The ITS-2 secondary structure also showed four helices (see Mai and Coleman [24]). Compared with ITS-1, the differences among the helices of ITS-2 were distributed across Helix I, II and IV. The ITS-2/CBC approach used for *Stichococcus*-like organisms presented in Pröschold and Darienko [11] revealed that both species differed from each other due to 12 changes (one CBC; two HCBCs; and nine deleted, single or unpaired bases). These findings supported that SAG 84.81 represents a new species, which will be described below.

**Figure 4:**
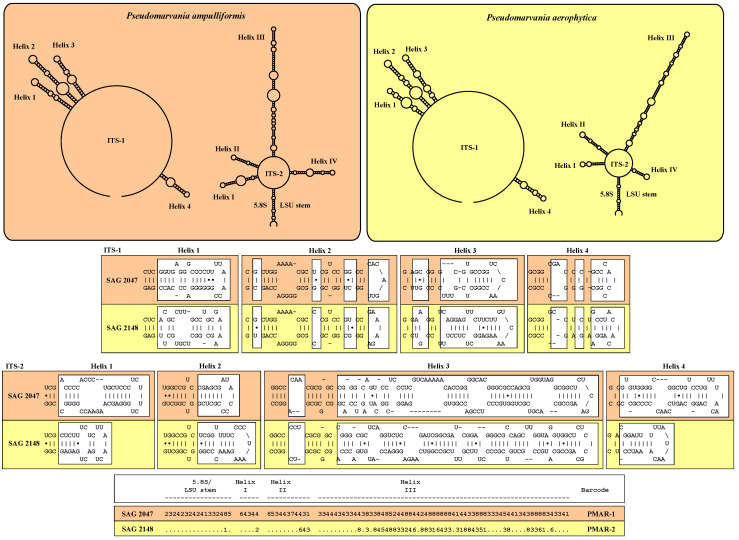
ITS-1 and ITS-2 secondary structures of the *Pseudomarvania* strains investigated in this study. The line structures have been drawn with PseudoViewer (http://pseudoviewer.inha.ac.kr). The helices were folded with mfold (http://www.unafold.org/RNA_form.php). The white boxes indicate the differences between both species. The conserved region of ITS-2 was used as barcode using the ITS-2/CBC approach as demonstrated in Pröschold and Darienko [11]. The ITS-2 Barcode is given as number code (1 = A–U; 2 = U–A; 3 = G-C; 4 = C–G; 5 = G•U; 6 = U•G; 7 = mismatch; 8 = deletion, single or unpaired bases). The species are highlighted in the different colors.

The ITS-1 and ITS-2 secondary structures of both *Pseudomarvania* species had the same four helix structures as shown for *Prasiolopsis*. However, both strains differed in more regions. Mainly, only the bases of each helix were almost constant between them. The ITS-2/CBC approach demonstrated that the recognition of two separate species is confirmed by eight CBCs, four HCBCs and thirty deleted, single or unpaired bases (Figure 4).

### 2.2. Morphology and Phenotypic Plasticity of the Investigated Prasiolopsis Strains

**Figure 5:**
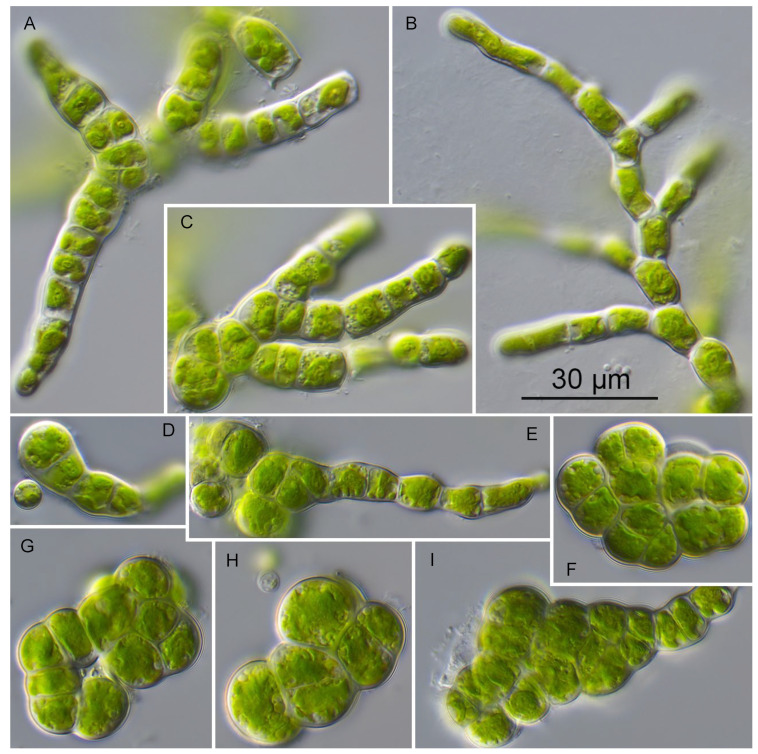
Morphology and phenotypic plasticity of *Prasiolopsis ramosa* ((**A**–**C**) SAG 2495 and (**D**–**I**) SAG 26.83) on 3N-BBM+V medium. (**A,B**). Young uniseriate branched filaments; (**C**). Filaments with cells becoming spherical; (**D**). Filaments becoming multiseriate; (**E**–**I**). Filaments forming cell packages. Scale bar = 30 μm.

The strains SAG 26.83, SAG 2495 and PR-1 showed an identical morphology, which was also described by Vischer [1] and documented by Kornmann and Sahling [10] for *Prasiolopsis ramosa*. The young thallus formed uniseriate short filaments, which can branch. The old thalli became multiseriate forming cell packages that are arranged into pseudoparenchyms. The chloroplasts were asteroid with a central pyrenoid. Cells were cylindrical with a cell length of 15–25 µm and a cell width of 7–9 µm (Figure 5). In contrast, the strain SAG 84.81 had smaller cell sizes and different cell shapes. The detailed description of the morphology is given in the following description of the new species of *Prasiolopsis*.

**Figure 6:**
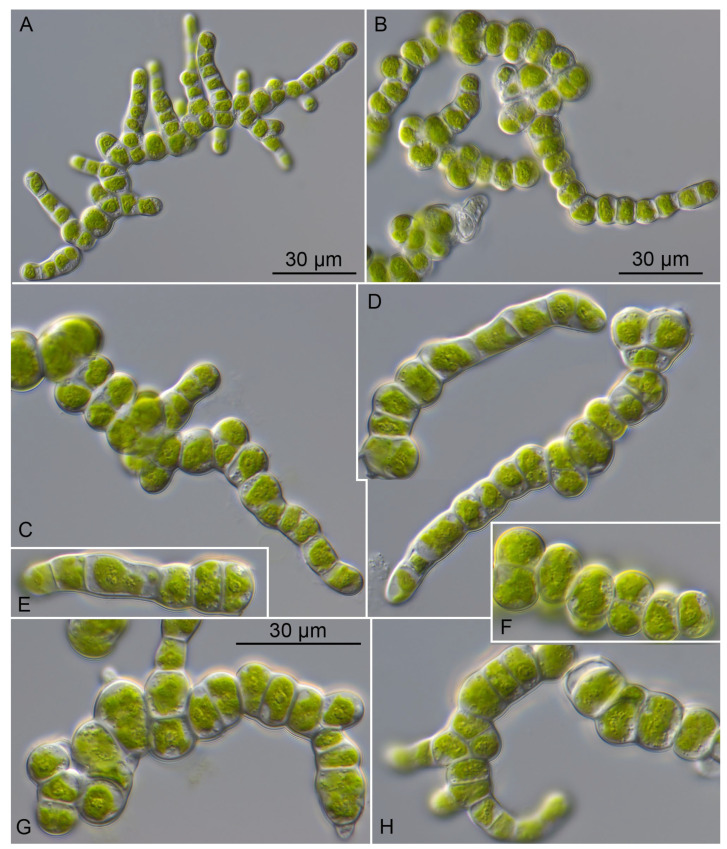
Morphology and phenotypic plasticity of *Prasiolopsis wulf-kochii* (SAG 84.81) on 3N-BBM+V medium. (**A**) Young uniseriate branched filaments; (**B**) filaments with cells becoming spherical; (**C**) filaments becoming multiseriate; (**D**–**H**) filaments forming cell packages. Scale bar = 30 μm.

***Prasiolopsis wulf-kochii*** sp. nov. (Figure 6)

*Description*: Young thalli consist of short-branched filaments. Later cells divide longitudinally and latitudinally, which lead to formation of bi-serial filaments or cubical cell packages and become pseudoparenchymatic. The cells in filaments are short, cylindrical, quadratic, sometimes almost spherical and constricted. The end cells of filaments are ovoid, sometimes pointed, or slightly curved. The cell size of cylindrical cells varies from 6.8 × 7.7 to 7.9 × 17.6 µm. Cells in packages are spherical and compressed from sites. Isodiametric cells are 6.8 × 7.5 to 10.2 × 14.9 µm. Compressed cells are 4.3 × 7.2 to 6.2 × 13.9 µm. Young cells have relatively thin cell walls of 0.5 µm, which become thicker with age up to 1.0 µm. Vegetative cells possess asteroid chloroplasts with a pyrenoid surrounded by many starch grains. Asexual reproduction occurs by vegetative division and aplanospore formation. No motile stages were observed.

*Diagnosis*: Differs from *P. ramosa* by having a smaller cell size and ecological distribution as well as its SSU+ITS sequences (GenBank: PQ108893) and ITS-2 Barcode: PR-2 in Figure 2.

*Holotype* (designated here): The strain SAG 84.81 has been permanently cryopreserved in a metabolically inactive state at the Culture Collection of Algae (SAG), University of Göttingen, Germany.

*Etymology*: The species is named in honor of Dr. Wulf Koch who was the curator of the Culture Collection of Algae (SAG) at the University of Göttingen (1954–1979). He isolated this strain in 1977 from the hairs of the sloth *Bradypus tridactylus.*

## 3. Discussion

### 3.1. Systematics, Ecology and Geographical Distribution of the Genus Prasiolopsis

In our study, the genus *Prasiolopsis* was found to contain two species: *P. ramosa* and the newly described *P. wulf-kochii*. As demonstrated, both species differed in terms of their morphology and molecular signatures, such as secondary structures of ITS-1 and ITS-2 and ITS-2 barcode with CBCs and HCBCs (Figure 1, Figure 2, Figure 3, Figure 5 and Figure 6). In addition, the strains SAG 26.83 and SAG 84.81 also showed differences in two plastid-coding genes, *rbc*L and *tuf*A, which are available in GenBank [15,16,18]. Both strains differed in three amino acids in *rbc*L (22 base differences between EF203015 for SAG 26.83 to KM462862 for SAG 83.81) and two in *tuf*A (21 base differences between LN877828 and KM462862), which supported the separation into two species. *P. wulf-kochii* was originally denoted as *Trichophilus welckeri*, a species occurring in or on the hairs of sloths. However, the presence of an asteroid chloroplast with a central pyrenoid and the absence of zoospores (typical for *Prasiolopsis*) contradicted this identification. *Trichophilus* had parietal chloroplasts without pyrenoid and formed two types of zoospores [20]. Both *Prasiolopsis* species have different ecological preferences. *P. ramosa* have been found on barks of trees [1,2,3], supralittoral of coast lines [18], marble monuments [25], granite outcrops [26] and granite walls, whereas *P. wulf-kochii* is only known from hairs of sloths (this study). Despite these few records, *Prasiolopsis* seemed to have a worldwide distribution, however, its recognition is probably caused by difficulties of identification based solely on morphology, as already mentioned by Vischer [1]. Das [27] reported *Prasiolopsis ramosa* from a sub-alpine waterfall of the Eastern Himalaya (India), but the provided micrographs in their study were of poor quality. Therefore, the identification is questionable because no morphological features were clearly visible. By checking the records in the GBIF database (https://www.gbif.org; accessed on 5 August 2024), 50 entries of *Prasiolopsis* have been reported from around the world (Europe, Australia, USA, Greenland and Antarctica). Unfortunately, these records cannot be assigned at the species level because these metabarcoding studies focused on topics other than Prasiolales and its relatives and there are only a few sequencing reads assigned as *Prasiolopsis* that cannot be proven because the sequencing data are not accessible. Further studies are necessary to decide if *Prasiolopsis* is a rare genus or merely overlooked by environmental studies.

Interestingly, *Pseudomarvania*—a genus with different morphology and reproduction—belongs to the family Prasiolaceae with the genera *Prasiola*, *Rosenvingiella*, *Prasiococcus* and *Prasiolopsis*. The two species of *Pseudomarvania* had a coccoid-to-rod-like morphology, and consisted of single, parietal, cup-shaped chloroplasts. Both taxa reproduce by budding, which is unique among all specimens belonging to the *Prasiola* clade [28,29,30].

### 3.2. The Mystery of Trichophilus Welckeri—A New View on an Old Story

As mentioned above, the strain SAG 84.81 was originally assigned as *Trichophilus welckeri*. Our study clearly revealed that this strain represents a new species of *Prasiolopsis*. This raises the question: What is *Trichophilus*? Two species of this genus have been described: *T. welckeri* Weber-van-Bosse [20] and *T. neniae* Lagerheim [31]. Both are epizoic on or in the hairs of different species of sloths and the shells of the freshwater snail *Nenia*, respectively. Since that time, epizoic algae have been the subject of different studies. Parasitic, symbiotic, and mutualistic interactions between vertebrates-algae are summarized in several recent reviews [32,33,34,35]. The algae–sloth interaction was of special interest. Pauli et al. [35] and Kaup et al. [33] referred to this as “mobile ecosystems”. Suutari et al. [21] studied the biomes on several sloth species using molecular methods. They discovered that many different eukaryotic algae lived in or on the hairs of the six investigated sloth species. These findings were confirmed by Kaup et al. [33]. Interestingly, both studies found one lineage, which they identified as *Trichophilus welckeri*. Phylogenetic analyses revealed that it belonged to the Ulvophyceae. Unfortunately, no cultured material of this species is available, and the morphological comparison presented in Suutari et al. [21] with the original description by Weber-van-Bosse [20] did not clearly demonstrate the identity of *Trichophilus*. For clarification of the taxonomic status of *Trichophilus*, it is necessary to isolate clonal cultures from the hairs of sloths, which then need to be investigated by the integrative approach used in this study. Despite the uncertain status of *Trichophilus*, the phylogenetic analyses of Suutari et al. [21] revealed that the so-called *Trichophilus* belonged to the Ulvophyceae without clear affiliation because of lack of data at that time. To find out if the three clades A, B and C belong to another described genus of the Ulotrichales or represent their own lineage within this order, we reanalyzed the SSU rDNA of these clades together with all representatives of this order.

**Figure 7:**
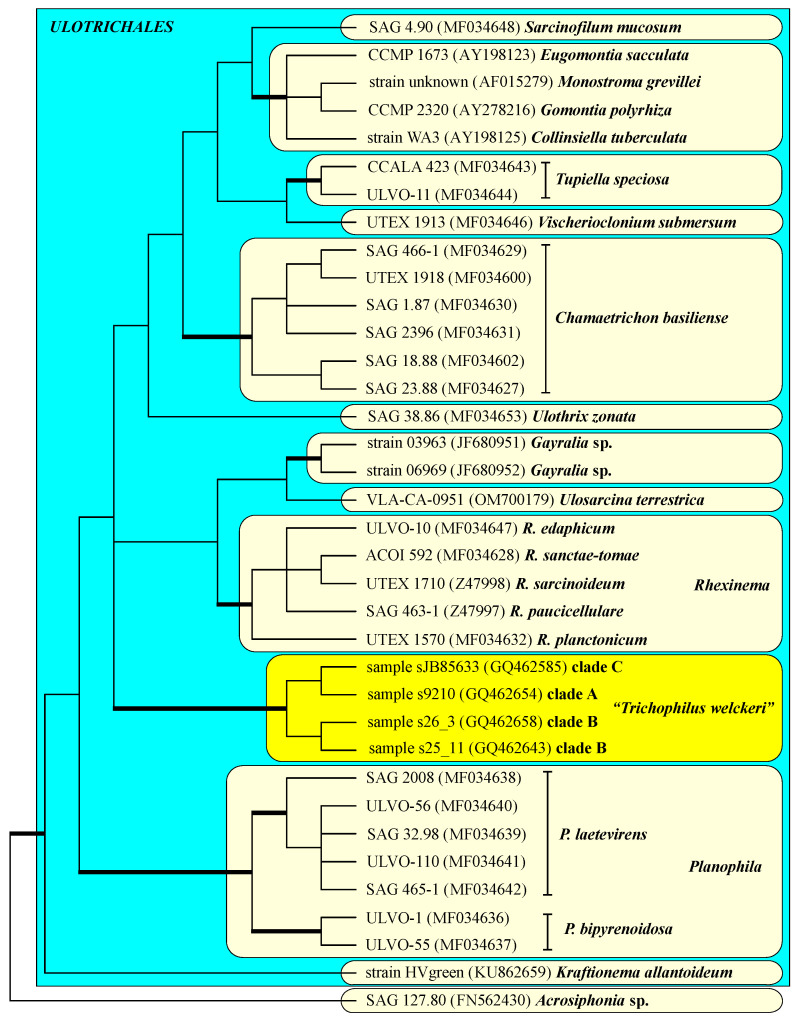
Molecular phylogeny of the *Ulotrichales* (Ulvophyceae) based on SSU rDNA sequence comparisons. The phylogenetic tree shown was created using the maximum likelihood method based on a data set of 1772 aligned positions of 36 taxa using PAUP 4.0a build169. For the analysis, the TVM+I+G (base frequencies: A 0.24566, C 0.21777, G 0.27468, U 0.26189; rate matrix A-C 0.6147, A-G 4.0595, A-U 0.7746, C-G 0.4119, C-U 4.0595, G-U 1.0000) with the proportion of invariable sites (I = 0.7737) and gamma shape parameter (G = 0.7167) was chosen, which was calculated as the best model by automated model selection tool implemented in PAUP. The branches in bold are highly supported in all analyses (Bayesian values >0.95 calculated with PHASE and MrBayes; bootstrap values >70% calculated with PAUP using maximum likelihood, neighbor-joining, maximum parsimony and RAxML using maximum likelihood). The sister order with *Acrosiphonia* was chosen as an outgroup.

Interestingly, the phylogenetic analyses clearly revealed that the sequences of Suutari et al. [21] represented their own lineage within the Ulotrichales, which is highly supported in all Bayesian and bootstrap analyses (Figure 7). Unfortunately, no ITS rDNA sequences of the three clades are available in GenBank. Therefore, it cannot be determined if these clades represent a species within this genus. It is also necessary to isolate cultures to determine which kind of interaction this green algal lineage has with the different species of sloths.

## 4. Materials and Methods

### 4.1. Cultures and Light Microscopy

All investigated strains, except for strain PR-1, originated from the Culture Collection of Algae (SAG) at the University of Göttingen, Germany (http://sagdb.uni-goettingen.de). Origins of the strains are listed as follows:

- SAG 26.83: Switzerland, Basel, bark of a fruit tree (47°28′52″ N, 7°51′7″ E); authentic strain of *Prasiolopsis ramosa* [1].
- SAG 2495: Germany, Hamburg, surface of a marble monument (53°33′43″ N, 9°59′27″ E); assigned as *Prasiolopsis* sp. [25].
- SAG 84.81: Brazil, Amazonia, from hairs of the sloth *Bradypus tridactylus*; assigned as *Trichophilus welckeri*.
- Strain PR-1: Austria, Vienna, Türkenschanzpark, wall near Dänenstrasse (48°14′8″ N, 16°20′2″ E); assigned as *Prasiolopsis ramosa*.
- For comparison, two strains of the sister genus *Pseudomarvania* were investigated:
- SAG 2047: Japan, Hiroshima Prefecture, Taishaku-kyo Gorge, bark on *Cephalotaxus harringtonia* (34°51′0″ N, 133°14′1″ E), authentic strain of *Pseudomarvania ampulliformis* [28,29].
- SAG 2148: Malaysia, Peninsular Malaysia, Hulu Kelantan, lowland rainforest, bark on *Pandanus* sp. (5°13′10″ N, 101°48′00″ E), authentic strain of *Pseudomarvania aerophytica* [29,30].

All strains were cultivated at 18 °C, with 50 μmol photons/m^2^s provided by daylight fluorescent tubes (Osram L36 W/954 Lumilux de lux daylight, Munich, Germany), and a light–dark cycle of 16:8 hrs on agarized modified Bold’s Basal Medium (3N-BBM+V; medium 26a in Schlösser [36]). The light microscopic investigations were conducted using an Olympus BX-60 microscope (Olympus, Tokyo, Japan), and the micrographs were taken with a ProgRes C14plus camera using the ProgRes CapturePro imaging system ((version 2.9.0.1), both from Jenoptik, Jena, Germany).

### 4.2. DNA Extraction, PCR, Sequencing and Phylogenetic Analyses

The genomic DNA of the strains was extracted using the DNeasy Plant Mini Kit (Qiagen, Hilden, Germany) and following the instructions provided by the manufacturer. The SSU and ITS rDNA were amplified in PCR reactions using the Taq PCR MasterMix Kit (Qiagen, Hilden, Germany) with two primer combinations EAF3/G800R and G500F/ITS055R [37]. All PCR products were purified and sequenced as described by Darienko et al. [37]. The sequences are available in the EMBL, GenBank and DDBJ sequence databases under the accession numbers given in Figure 1.

The SSU rDNA sequences of all strains were aligned according to their secondary structures. The ITS-1 and ITS- 2 sequences of all strains were folded according to the protocol described in detail in Pröschold and Darienko [11]. The alignment was included in a data set of 25 SSU and ITS rDNA sequences of *Prasiola* clade (2671 bp).

We used the Automated Model Selection tool implemented in PAUP* version 4.0a (build 169; [38]) for the decision about which evolutionary model best fit the data set. The settings of the best model are given in the legend of Figure 1. EMBL/GenBank accession numbers of published sequences and strain designations are provided in both of these figures. Phylogenetic trees were constructed using distance, parsimony and maximum likelihood criteria using PAUP [38], and the robustness of the tree topologies was proven with different Bayesian and bootstrap analyses (1000 replicates). In addition, the programs RAxML version 8.2.12 [39], MrBayes version 3.2.7a [40] and PHASE package 2.0 [41,42,43,44,45] were used. 

The secondary structures of ITS-1 and ITS-2 sequences were folded using the computer programs mfold [46] and visualized using the web-based program PseudoViewer3 (http://pseudoviewer.inha.ac.kr; [47]).

## Data Availability

Data are contained within the article.

## References

[1] Vischer W. (1953). Über primitivste Landpflanzen. Ber. Schweiz. Bot. Ges..

[2] Chodat R. (1909). Etude Critique et Expérimentale sur le Polymorphisme des Algues.

[3] Brand F. (1925). Analyse der aerophilen Grünalgenanflüge, insbesondere der proto-pleurococcoiden Formen. Arch. Protistenkd..

[4] Schaffner J.H. (1922). The classification of plants XII. Ohio J. Sci..

[5] Knebel G. (1935). Monographie der Algenreihe der Prasiolales, insbesondere von *Prasiola crispa*. Hedwigia.

[6] Silva P.C. (1957). Notes on Pacific marine algae. Madroño.

[7] Friedmann I. (1959). Structure, life-history and sex determination of *Prasiola stipitata* Suhr. Ann. Bot..

[8] Friedmann I. (1969). Geographic and environmental factors controlling life history and morphology in *Prasiola stipitata* Suhr. Österr. Bot. Z..

[9] Friedmann I., Manton I. (1960). Gametes, fertilization and zygote development in *P. stipitata*. Nova Hedwigia.

[10] Kornmann P., Sahling P.H. (1974). Prasiolales (Chlorophyta) von Helgoland. Helgol. Wiss. Meeresunters..

[11] Pröschold T., Darienko T. (2020). The green puzzle *Stichococcus* (Trebouxiophyceae, Chlorophyta): New generic and species concept among this widely distributed genus. Phytotaxa.

[12] O’Kelly C.J., Garbary D.J., Floyd G.L. (1989). Flagellar apparatus of male gametes and other aspects of gamete and zygote ultrastructure in *Prasiola* and *Rosenvingiella* (Chlorophyta, Prasiolales) from British Columbia. Can. J. Bot..

[13] Sherwood A.R., Garbary D.J., Sheath R.G. (2000). Assessing the phylogenetic position of the Prasiolales (Chlorophyta) using *rbc*L and 18S rRNA sequence data. Phycologia.

[14] Karsten U., Friedl T., Schumann R., Hoyer K., Lembcke S. (2005). Mycosporine-like amino acids and phylogenies in green algae: *Prasiola* and its relatives from the Trebouxiophyceae (Chlorophyta). J. Phycol..

[15] Rindi F., McIvor L., Sherwood A.R., Friedl T., Guiry M.D., Sheath R.G. (2007). Molecular phylogeny of the green algal order Prasiolales (Trebouxiophyceae, Chlorophyta). J. Phycol..

[16] Heesch S., Pažoutová M., Moniz M.B.J., Rindi F. (2016). Prasiolales (Trebouxiophyceae, Chlorophyta) of the Svalbard Archipelago: Diversity, biogeography and description of the new genera *Prasionella* and *Prasionema*. Eur. J. Phycol..

[17] Darienko T., Gustavs L., Pröschold T. (2016). Species concept and nomenclatural changes within the genera *Elliptochloris* and *Pseudochlorella* (Trebouxiophyceae) based on an integrative approach. J. Phycol..

[18] Hodac L., Hallmann C., Spitzer K., Elster J., Faßhauer F., Brinkmann N., Lepka D., Diwan V., Friedl T. (2016). Widespread green algae *Chlorella* and *Stichococcus* exhibit polar-temperate and tropical-temperate biogeography. FEMS Microbiol. Ecol..

[19] Lemieux C., Otis C., Turmel M. (2014). Chloroplast phylogenomic analysis resolves deep-level relationships within the green algal class Trebouxiophyceae. BMC Evol. Biol..

[20] Weber-van-Bosse A. (1887). Étude sur les algues parasites des Paresseux. Natuurk. Verh. Holl. Maatsch. Wetensch. Haarlem III.

[21] Suutari M., Majaneva M., Fewer D.P., Voirin B., Aiello A., Friedl T., Chiarello A.G., Blomster J. (2010). Molecular evidence for a diverse green algal community growing in the hair of sloths and a specific association with *Trichophilus welckeri* (Chlorophyta, Ulvophyceae). BMC Evol. Biol..

[22] Altschul S.F., Gish W., Miller W., Myers E.W., Lipman D.J. (1990). Basic local alignment search tool. J. Mol. Biol..

[23] Coleman A.W., Mai J.C. (1997). Ribosomal DNA ITS-1 and ITS-2 sequence comparisons as a tool for predicting genetic relatedness. J. Mol. Evol..

[24] Mai J.C., Coleman A.W. (1997). The internal transcribed spacer 2 exhibits a common secondary structure in green algae and flowering plants. J. Mol. Evol..

[25] Hallmann C., Rüdrich J., Enseleit M., Friedl T., Hoppert M. (2011). Microbial diversity on a marble monument: A case study. Environ. Earth Sci..

[26] Mikhailyuk T.I. (2013). Terrestrial Algae from the Granite Outcrops of River Valleys of the Ukraine. Internat. J. Algae.

[27] Das S.K. (2015). *Prasiolopsis ramosa* Vischer—New addition to the algal flora of Asia. J. New Biol. Rep..

[28] Handa S., Nakahara M., Tsubota H., Deguchi H., Nakano T. (2003). A new aerial alga, *Stichococcus ampulliformis* sp. nov. (Trebouxiophyceae, Chlorophyta) from Japan. Phycol. Res..

[29] Elias M., Neustupa J. (2009). *Pseudomarvania*, gen. nov. (Chlorophyta, Trebouxiophyceae), a new genus for “budding” subaerial green algae *Marvania aerophytica* Neustupa et Sejnohova and *Stichococcus ampulliformis* Handa. Fottea.

[30] Neustupa J., Sejnohova L. (2003). *Marvania aerophytica* sp. nov., a new subaerial tropical green alga. Biologia.

[31] Lagerheim G. (1892). *Trichophilus neniae* Lagerh. n. sp., eine neue epizoische Alge. Ber. Dt. Bot. Ges..

[32] Yang H., Genot B., Duhamel S., Kerney R., Burns J.A. (2022). Organismal and cellular interactions in vertebrate–alga symbioses. Biochem. Soc. Trans..

[33] Kaup M., Trull S., Hom E.F.Y. (2021). On the move: Sloths and their epibionts as model mobile ecosystems. Biol. Rev..

[34] Fountain E.D., Pauli J.N., Mendoza J.E., Carlson J., Peery M.Z. (2017). Cophylogenetics and biogeography reveal a coevolved relationship between sloths and their symbiont algae. Mol. Phylogen. Evol..

[35] Pauli J.N., Mendoza J.E., Steffan S.A., Carey C.C., Weimer P.J., Peery M.Z. (2022). A syndrome of mutualism reinforces the lifestyle of a sloth. Proc. R. Soc. B.

[36] Schlösser U.G. (1997). Additions to the culture collections of algae since 1994. Bot. Acta.

[37] Darienko T., Rad Menéndez C., Campbell C., Pröschold T. (2019). Are there any true marine *Chlorella* species? Molecular phylogenetic assessment and ecology of marine *Chlorella*-like organisms, including description of *Droopiella* gen. nov. Syst. Biodivers..

[38] Swofford D.L. (2002). PAUP* Phylogenetic Analysis Using Parsimony (*and Other Methods).

[39] Stamatakis A. (2014). RAxML version 8: A tool for phylogenetic analysis and post-analysis of large phylogenies. Bioinformatics.

[40] Ronquist F., Teslenko M., Van Der Mark P., Ayres D.L., Darling A., Höhna S., Larget B., Liu L., Suchard M.A., Huelsenbeck J.P. (2012). MrBayes 3.2: Efficient Bayesian phylogenetic inference and model choice across a large model space. Syst. Biol..

[41] Jow H., Hudelot C., Rattray M., Higgs P. (2002). Bayesian phylogenetics using an RNA substitution model applied to early mammalian evolution. Mol. Biol. Evol..

[42] Higgs P., Jameson D., Jow H., Rattray M. (2003). The evolution of tRNA-*Leu* genes in animal mitochondrial genomes. J. Mol. Evol..

[43] Hudelot C., Gowri-Shankar V., Jow H., Rattray M., Higgs P. (2003). RNA-based phylogenetic methods: Application to mammalian mitochondrial RNA sequences. Mol. Phylogen. Evol..

[44] Gibson A., Gowri-Shankar V., Higgs P., Rattray M. (2005). A comprehensive analysis of mammalian mitochondrial genome base composition and improved phylogenetic methods. Mol. Biol. Evol..

[45] Telford M.J., Wise M.J., Gowri-Shankar V. (2005). Consideration of RNA secondary structure significantly improves likelihood-based estimates of phylogeny: Examples from the bilateria. Mol. Biol. Evol..

[46] Zuker M. (2003). Mfold web server for nucleic acid folding and hybridization prediction. Nucleic Acid Res..

[47] Byun Y., Han K. (2009). PseudoViewer3: Generating planar drawings of large-scale RNA structures with pseudoknots. Bioinformatics.

